# Achieving optimal cancer outcomes in East Africa through multidisciplinary partnership: a case study of the Kenyan National Retinoblastoma Strategy group

**DOI:** 10.1186/s12992-016-0160-1

**Published:** 2016-05-26

**Authors:** Jessica A. Hill, Kahaki Kimani, Abby White, Faith Barasa, Morgan Livingstone, Brenda L. Gallie, Helen Dimaras, Daisy’s Eye Cancer Fund & The Kenyan National Retinoblastoma Strategy Group

**Affiliations:** Department of Ophthalmology and Vision Sciences, The Hospital for Sick Children, 555 University Ave., Room 7265, Toronto, ON M5G 1X8 Canada; Department of Ophthalmology, University of Nairobi, Nairobi, Kenya; World Eye Cancer Hope (formerly Daisy’s Eye Cancer Fund - International), http://www.wechope.org/; Daisy’s Eye Cancer Fund-Kenya, Nairobi, Kenya; Department of Ophthalmology & Vision Sciences, Faculty of Medicine & Division of Clinical Public Health, Dalla Lana School of Public Health, University of Toronto, Toronto, Canada; Division of Clinical Public Health, Dalla Lana School of Public Health, University of Toronto, Toronto, Canada; Department of Human Pathology, University of Nairobi, Nairobi, Kenya

**Keywords:** Multidisciplinary, Multisectoral, Partnership, Kenya, Retinoblastoma, Global health, Capacity building

## Abstract

**Background:**

Strategic, interdisciplinary partnerships are essential to addressing the complex drivers of health inequities that result in survival disparities worldwide. Take for example the aggressive early childhood eye cancer retinoblastoma, where survival reaches 97 % in resource-rich countries, but is as low 30 % in some resource-limited nations, where 92 % of the burden lies. This suggests a need for a multifaceted approach to achieve a tangible and sustainable increase in survival.

**Methods:**

We assembled the history the Kenyan National Retinoblastoma Strategy (KNRbS), using information documented in NGO reports, grant applications, news articles, meeting agendas and summaries. We evaluated the KNRbS using the principles found in the guide for transboundary research partnerships developed by the Swiss Commission for Research Partnerships with Developing Countries.

**Results:**

A nationally co-ordinated approach drawing input and expertise from multiple disciplines and sectors presented opportunities to optimise cure of children with retinoblastoma. Annual meetings were key to achieving the over 40 major outputs of the group’s efforts, related to Awareness, Medical Care, Family Support and Resource Mobilization. Three features were found to be critical to the KNRbS success: multidisciplinarity, consistency and flexibility.

**Conclusion:**

The KNRbS has achieved a number of key outputs with limited financial investment. As a partnership, the KNRbS meets most of the criteria identified for success. Challenges remain in securing the long-term sustainability of its achievements. Elements of the Kenyan National Retinoblastoma Strategy may be useful to other developing countries struggling with limited survival of retinoblastoma and other cancers or rare diseases.

**Electronic supplementary material:**

The online version of this article (doi:10.1186/s12992-016-0160-1) contains supplementary material, which is available to authorized users.

## Background

National strategies are a relatively recent innovation in sustainable development. They mark a transition from a fixed strategy towards an adaptive one that allows for continuous improvement [1]. National strategies have become increasingly popular in addressing healthcare challenges. It has been noted that a well-conceived, well-orchestrated national strategy can reduce cancer incidence and improve the quality of life for persons with cancer [2].

To be effective, a national strategy requires investment and expertise from partners across multiple disciplines, providing critical information on how best to deliver healthcare, enact policy and effectively evaluate processes. Partnerships between actors in resource-rich and resource-limited nations which are mutually beneficial are becoming increasingly common. Here we describe an international, multidisciplinary partnership conceived to improve survival in Kenya from a rare, aggressive childhood eye cancer, retinoblastoma.

An estimated 760 children are newly affected by retinoblastoma annually in the 11 countries constituting East Africa [3]. Outcomes for retinoblastoma in East Africa have been poor, with survival estimated at 23 % in 2006 [4]. Kenya has a relatively large burden of children with retinoblastoma and historically low levels of survival, but also a group of individuals willing to work together to improve care. These factors provided an impetus for developing the Kenyan National Retinoblastoma Strategy (KNRbS), a multidisciplinary, multisectoral partnership aimed at improving retinoblastoma outcomes in Kenya.

Delivering optimal care for retinoblastoma is complex and requires a multidisciplinary team. This team includes medical specialists, such as an ophthalmologist, oncologist, pathologist and ideally a geneticist [5, 6]. The medical specialists, in turn, rely on technical experts like imaging specialists to be able to accurately diagnosis and treat patients. Furthermore, social workers, child life experts and genetic counsellors sensitively and accurately disseminate information to a family affected by retinoblastoma, which is critical for making the best management choices. Assembling such a team is challenging in any situation, and particularly in low-resource settings. Additional challenges to delivering best care for patients with retinoblastoma include limited health-seeking behaviour due to cultural factors or lack of public awareness; socioeconomic barriers to healthcare access; and weak or immature health care infrastructure and health systems that limits availability of care [7, 8]. Increasingly, global efforts to improve retinoblastoma survival are being made to address these various barriers to care (reviewed in [9]).

In this article, we discuss the conception and implementation of the KNRbS, and examine milestones the group has achieved and challenges encountered. More broadly, we explore how national strategies may serve to improve cancer survival in resource-limited countries.

## Methods

### Historical analysis

The history and goals of the Kenyan National Retinoblastoma Strategy (KNRbS) were assembled using information documented in NGO reports, grant applications, news articles, meeting agendas and summaries. The number, frequency, attendance, and topics covered at annual KNRbS meetings were documented. Key activities and outputs were listed and organized by relationship to overall goals of Awareness, Medical Care, Family Support and Resource Mobilization.

### Conceptual framework

Several frameworks exist for the evaluation the success of partnerships. We evaluated the KNRbS using the principles found in the guide for transboundary research partnerships developed by the Swiss Commission for Research Partnerships with Developing Countries (KFPE) [10]. The KFPE framework was developed specifically for research partnerships. Although the goal of the KNRbS strategy was not solely to expand research capacity, it was recognized early on that solid data collection, and careful measurement and application of the scientific method would be key to evaluating impact, and thus research has played a central role in its development.

## Results

### The KNRbS: a brief history

The creation of the KNRbS was driven by the knowledge that survival from retinoblastoma is possible, and buoyed by Daisy’s Eye Cancer Fund (DECF), an NGO with the mandate to save lives and vision for children affected by retinoblastoma. The discrepancy in retinoblastoma survival between resource-rich and resource-poor countries is highlighted by the event that spurred DECF to focus on retinoblastoma in Sub-Saharan Africa. As a baby, Rati was diagnosed with retinoblastoma in her home country of Botswana and had the affected eye removed at 11 months of age. Histopathological analysis showed high-risk features necessitating further treatment, however this report did not make it to the treating physician in time. Rati developed cancer recurrence and sought treatment in Canada, but ultimately died at age four. Her death sparked the creation of *Rati’s Challenge*, a major focus of DECF aimed at addressing poor retinoblastoma survival in Sub-Saharan Africa [11].

The primary geographical focus for *Rati’s Challenge* quickly became Kenya for several reasons. Kenya experiences a significant number of new retinoblastoma cases per year (~90), and Nairobi is a key ophthalmic hub in East Africa. Crucially, the DECF team visited Kenya and met with several highly motivated individuals that shared their goals of increasing retinoblastoma survival in East Africa [11].

Initial meetings in 2006 with Kenyan clinicians, retinoblastoma survivors and community leaders indicated a desire to create a formal national effort. In 2007, DECF members visited four major retinoblastoma treatment centres in Kenya (Nairobi, Eldoret, Kisumu and Mombasa) and met with various stakeholders, leading to the emergence of the KNRbS group. The focus of this group was primarily to create a national strategy to improve retinoblastoma outcomes. The group also aimed to serve as a template for the creation of national strategies in neighbouring countries. A Kenyan chapter of DECF was established and registered as an NGO in Nairobi to plan and execute activities related to the strategy. This ensured the partnership was locally-led and locally-driven.

### The KNRbS group

**Fig. 1:**
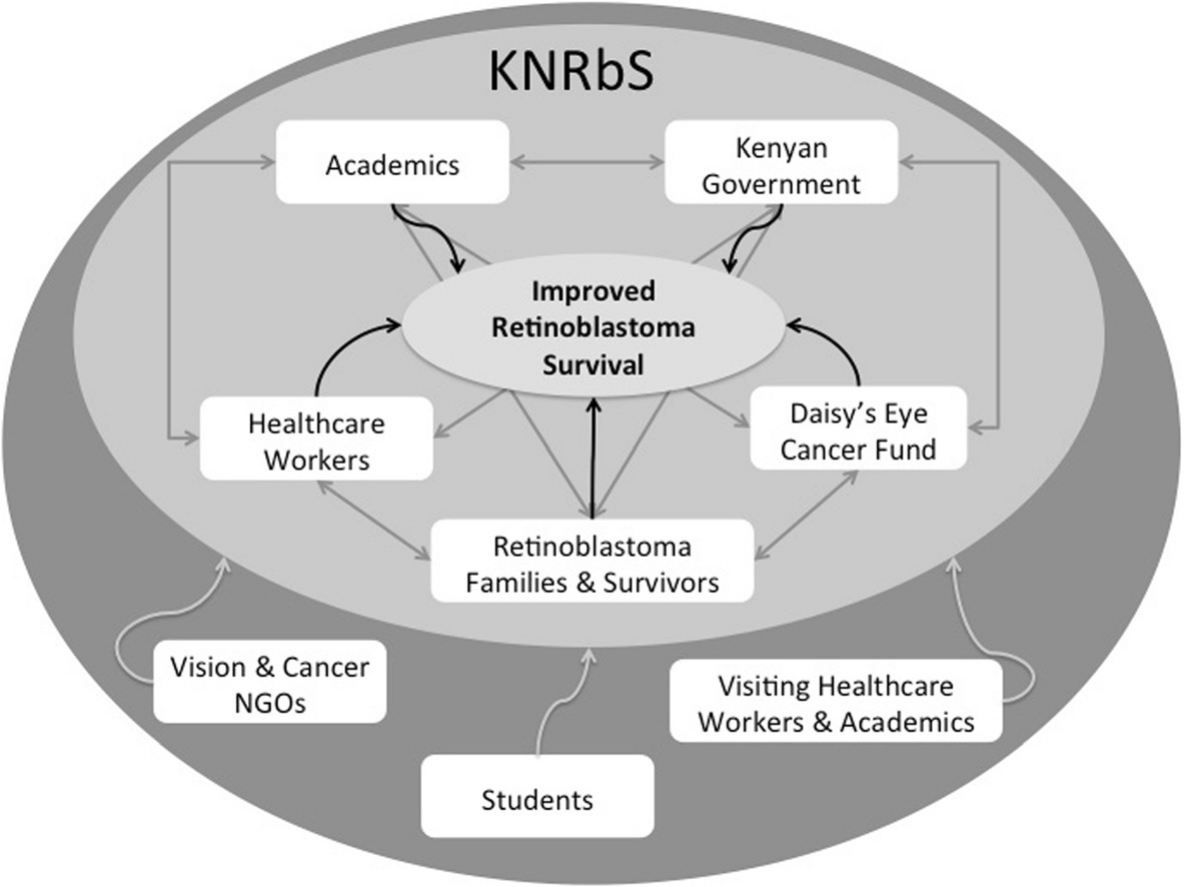
KNRbS Membership. The KNRbS group (inner, light gray oval) is made up of a multidisciplinary team of academics (researchers, professors/educators), members of healthcare teams (oncologists, ophthalmologists, nurses, pathologists, lab technicians, child life leaders, ophthalmic clinical officers), government officials (Ministry of Medical Services, Department of Ophthalmic Services staff), staff and board members of Daisy’s Eye Cancer Fund, retinoblastoma survivors and families. Communication and collaboration (grey arrows) between these groups allows for tangible contributions towards improving retinoblastoma survival (black arrows). Through the KNRbS annual meetings, special ‘guests’ have attended and participated on an ad-hoc basis (outer, dark gray oval), and have included members of Vision and Cancer NGOs, students participating on short-term research projects, and visiting healthcare workers from East and West Africa

The membership of the KNRbS group is wide-ranging and multidisciplinary. Members are united by their professional and/or personal commitment to retinoblastoma, and include healthcare workers (e.g. ophthalmologists, oncologists, nurses, ophthalmic clinical officers, child life practitioners), academics (e.g. university professors, researchers), retinoblastoma survivors and their families, members of Kenyan government, and staff and board members of DECF (Fig. 1). Most are Kenyan, hailing from all over the country; few (~5) members are from UK and Canada.

The membership was purposely designed to be multidisciplinary and multisectoral, to cultivate cross-talk and collaboration to break through the barriers of traditional ‘silos’ most professions operate within. Members from government agencies and DECF bring skills in diplomacy, partnership building, and outreach. Their involvement promotes integration and sustainability of KNRbS efforts to effect healthcare delivery. Complementing this, members from academia and healthcare bring medical expertise and strengths in research and evaluation. Within the healthcare group, the involvement of individuals from the multiple disciplines that provide retinoblastoma care [5] promotes a comprehensive approach to retinoblastoma management. Above all, the families and retinoblastoma survivors remind the KNRbS group that the central focus of all activities should remain on the patient. They contribute their lived experience to focus efforts on psychosocial challenges and basic human rights of patients.

### The KNRbS annual meetings

Injecting new ideas and fresh resolve, the lifeblood of the National Strategy is the working meetings of the KNRbS group. Since 2008, meetings have taken place annually, lasting roughly three days each, and are hosted in different Kenyan cities to facilitate participation from members in different regions (Table 1).

**Table 1:** Annual KNRbS Meetings and Outcomes

Meeting #	Year	Location	# of days	# of delegates	Overall theme	Primary aim(s)	Invited speakers
1	2008	Nairobi	3	58	Planning	• Identify current challenges and priorities in retinoblastoma management	
2	2009	Mombasa	3	68	Planning	• Create 5 year plan for KNRbS	Ophthalmology Society of East Africa: Access to services for the blind
						• Update on progress with respect to 4 common challenges	
						• Discussion of national collaborative laboratory pathology service (RbCoLab)	
						• Introduction to electronic medical database (eCancerCare-Retinoblastoma)	
3	2010	Nairobi	3	42	Capacity Building	• Discussion of adoption of KNRbS guidelines	Kenyan Cancer Association (KENCASA): Kenya Cancer Bill overview
						• Focus on electronic medical database	
4	2011	Mombasa	3	42	Capacity Building	• Skills enhancement: surgical techniques; child life practices	Anthropologist: Managing cancer in a Kenyan Hospital
						• Knowledge enhancement: retinoblastoma genetics; indications for chemotherapy; grant writing	US Journalist: UN Summit on Non Communicable Diseases
							Kenya Society for the Blind: Coping with Blindness
5	2012	Nairobi	2 + 1 (workshop preceding)	60	Capacity Building	• Conference preceded by 1 day clinical skills workshop	
						• Focus on long term sustainability of KNRbS	
						• Discussion of the adoption of the retinoblastoma guidelines	
6	2013	Eldoret	2 + 1 (workshop preceding)	80	Research, Innovation & Implementation	• Conference preceded by 1 day clinical skills workshop	Christian Blind Mission: Implementation of Guidelines
						• Focus on retinoblastoma genetics and pathology	East African Strategies - Uganda, Ethiopia
						• Discussion of logistics of guideline implementation	
7	2014	Nairobi	3	60	Research, Innovation & Implementation	• Focus on research	UoN Research Services
						• Introduction to international retinoblastoma group (One RB World)	Technology: IT and mobiles in health services and management

While the first meeting aimed to critically assess and document the retinoblastoma situation in Kenya and identify challenges and solutions to be addressed (see Situation analysis), subsequent meetings have functioned by promoting critical reflection and discussion of activities, encouraging multidisciplinary collaboration by working in real time, and ensuring documentation of outcomes and intended goals for the year ahead. A focus on measurement, evaluation and research has helped the group inch toward goals.

KNRbS members work in four thematic Task Forces: Awareness, Medical Care, Family Support and Resource Mobilization (Table 2). (For a thorough accounting of the achievements of the KNRbS organized by Task Force, see Additional file 1: Table S1.) Specialty working groups have arisen organically within these in order to hone in on specific challenges (e.g. Pathology and Genetics groups sprouted from Medical Care; a Child Life group from Family Support). The activities within these groups are fluid and may change each year as evidence and experience grows and members learn what is effective and what is not.

**Table 2:** KNRbS Task Forces & Achievements

Focus group	Challenge	Solutions achieved (Selected)
Awareness	• Low public awareness of retinoblastoma	• Posters, Radio, TV, Print Media
	• Low medical/healthcare professional awareness of retinoblastoma	• Workshops, Maternal Child Health Booklet
Medical Care	• Lack of data on retinoblastoma patient survival, referral, outcomes	• Research
	• Delayed referral of patients	• Consensus Best Practice Guidelines, Research
	• Poor availability of specialist care	• Training, Consensus Best Practice Guidelines
	• Delayed and inaccurate pathology	• RbCoLab - Health Service Delivery Innovation
Family Support	• Socioeconomic challenges in accessing healthcare	• Cycle of Light
	• Families have trouble coping with and understanding retinoblastoma	• Child Life Training Program
Resource Mobilization	• Little funding for programmatic support	• Grants, Fundraisers
	• Difficult to mobilize support and strategic partnerships for retinoblastoma	• KNRbS meetings and invited guests

The KNRbS group has benefited from the participation of ‘invited guests’ at the annual general meetings (Table 1, Fig. 1). The contribution of these guests is to provide training or to introduce a new perspective that may help advance goals. Guests have included a representative of the Kenya Cancer Association, who described his group’s experience working with government to introduce the Kenyan Cancer Bill; staff from a Kenyan NGO with expertise in implementation of health policy; and representatives from the University of Nairobi Research Services office (Table 1). Occasionally students from Canada and Kenya have joined the KNRbS meetings to contribute to research and capacity building efforts (Fig. 1).

In addition to attending the annual working meetings, subsets of the group occasionally meet informally between annual meetings to work towards agreed-upon goals. However, communication between meetings for much of the larger group has remained challenging, making the annual meetings essential to progress for the KNRbS as a whole. Administrative support and leadership from DECF-K has been necessary for the organization and hosting of the annual meetings.

The annual meetings were the only direct financial costs of the KNRbS partnership. Costs of attendance were covered for all participants (venue, accommodation, modest travel allowance). Grants and charitable donations (Additional file 1: Table S1) were obtained to support the meetings. The meetings also benefited from in-kind support from KNRbS membership (e.g. DECF-K staff provided administrative support and co-ordination; invited speakers volunteered their time and expertise, etc.). Costs related to the KNRbS achievements (next section) are considered outputs in the context of this analysis, as their purpose was not to directly support the KNRbS partnership.

### KNRbS achievements

From a high-level perspective, the activities of the KNRbS group have moved through 3 distinct stages: Planning, Capacity Building, and Research and Innovation (Table 1). We highlight key achievements here.

#### Situation analysis

Critical to the improvement of retinoblastoma outcomes in East Africa is the identification of challenges to survival, and resources available for retinoblastoma care. A situation analysis was performed in the first meeting of the KNRbS team in 2008 [12]. The major challenges to the survival of affected children were identified as low awareness, delayed referral, poor access to specialist care and pathology, and lack of family support. Another challenge was identified in terms of resources required to implement solutions. The challenges are listed by related Task Force in Table 2. Immediate goals included the development of a public awareness poster campaign, a national retinoblastoma pathology service, agreed-upon Best Practice Guidelines for Kenya, and training of healthcare workers in Child Life practices. To raise funds to support these programs, fundraising and applications to granting agencies were proposed.

#### National guidelines for retinoblastoma care

The publication of Kenyan National Retinoblastoma Strategy Best Practices Guidelines represents a major milestone reached by the KNRbS group in 2014 (Table 1). The Canadian National Retinoblastoma Strategy Guidelines for Care [6] were used as a model and adapted to the Kenyan context. Initially, each Task Force aimed to produce specific document chapters, with a large amount of work to be completed outside of the annual meeting. However, the group met communication challenges, technological limitations, particularly for collaborators in rural areas where Internet and computers were not easily accessed, and other responsibilities that distracted from this purpose. Instead, the entire group worked on writing and editing the guidelines at each KNRbS annual meeting from 2008–2013 (Table 1). Although this method was likely more time consuming, it ensured that all group members were able to contribute to guideline development. Group consensus on the guidelines was reached at the 2013 KNRbS meeting. At the 2014 meeting, the published guidelines were debuted by the Ministry of Health, and distributed to all health clinics. They are freely available online [13].

#### Awareness

The KNRbS initiated many efforts to increase retinoblastoma awareness, with much support from NGO and government members of the group (Additional file 1: Table S1). DECF-K published annual reports in the early years of the project to inform the public of retinoblastoma facts and figures and highlight KNRbS activities designed in response. DECF-K was also instrumental in making connections with television, radio and print media outlets, which ran stories featuring retinoblastoma patients and several KNRbS efforts (Additional file 1: Table S1). A Swahili-language retinoblastoma awareness poster designed by the KNRbS Awareness taskforce and approved by the KNRbS group was printed by DECF-K and distributed by the Kenya Ministry of Health in clinics throughout the country. This information is also presented in the Kenyan *Maternal and Child Health Booklet*, a nationally distributed paper record intended to track a child’s health and development. Additional awareness activities included presentations at national and international conferences by KNRbS members, and hosting retinoblastoma-themed rounds at the home institutions of KNRbS members.

#### Family support

The KNRbS recognized immediately the need for supportive care for families coping with a diagnosis of retinoblastoma. Facilitating access to government subsidized health insurance (National Health Insurance Fund, NHIF) helped ease financial burden to indirectly assist with meeting transportation and local accommodation costs (see Research Mobilization). Anecdotal evidence from physicians indicates this was accompanied by increased treatment and follow-up compliance.

To address the transportation barrier directly, the potential for a bus company to provide subsidized transport for retinoblastoma-affected families was investigated. However, companies were reluctant to commit due to concern that they would be overwhelmed by the level of need. Currently, small funds are provided to families for transport when they cannot pay, funded by donations from Kenyan community groups and individuals.

Another approach considered was the development of family accommodation, “Kito House”. A document was developed, detailing the needs of the planned facility. One major challenge identified was the cost of land in Nairobi and the practicality of transporting families from a site outside the city centre, where land is more affordable but transport more complex. Family Support task force members made an onsite visit to a successful facility for families of cancer patients, “Ujasiri House” in Dar es Salaam, Tanzania [14]. This house is funded by a nonprofit but is built on the site of a public hospital, on land leased by the hospital for 10 years. Meals and utilities are supplied and funded by the hospital. The KNRbS team and the Kenya Childhood Cancer Trust are now investigating the possibility of developing a similar house on land belonging to Kenyatta National Hospital in Nairobi. This requires careful, long term planning, and will be a major focus of the Family Support task force in the coming years.

#### Training

Several training endeavours have been initiated by the KNRbS, both with an individual focus (fellowships) and a group focus (workshops). Two KNRbS members were selected for international fellowships in Ocular & Retinoblastoma Pathology, and Clinical Retinoblastoma Practice, respectively. Workshops were generally linked to the KNRbS meetings (Table 1) to ensure optimal attendance and reduce costs. Topics included retinoblastoma genetics, myoconjunctival enucleation technique [15], introduction to an electronic retinoblastoma care database (see eCancerCare-Retinoblastoma), ophthalmology and oncology for retinoblastoma, grant writing and child life practices (Additional file 1: Table S1). One workshop concerning handling and processing of eye specimens for pathology technicians (Additional file 1: Table S1) took place outside the regular KNRbS meeting schedule. This was because the target participants fell outside the main KNRbS membership, yet their training was essential to the development of the retinoblastoma pathology service (see The Retinoblastoma Collaborative Laboratory - RbCoLab).

Training has extended to the collaborative supervision of two graduate students at the University of Nairobi. Supporting future researchers is an integral aspect of building research capacity in Kenya.

#### Resource mobilization

Resources were mobilized in the form of grants, private monetary and in-kind donations, and political support (Additional file 1: Table S1). The first KNRbS meeting was supported in part by a grant from the Canadian Institutes for Health Research (CIHR). Reporting back to the CIHR on this inaugural meeting provided a format for setting up measurable outcomes, supporting a research approach. Additional grant funds have been obtained since in support of annual meetings, training and innovations sprouting from the group. DECF has also experimented with event fundraising to support KNRbS endeavours, with modest success (Additional file 1: Table S1).

A rather innovative approach for alleviating the financial burden of care faced by families was a microfinance program, ‘Cycle of Light’, developed by DECF-K (Additional file 1: Table S1). Parents of inpatients with retinoblastoma were taught to make handicrafts, which were sold to cover costs associated with hospitalization, namely NHIF premiums, additional treatment costs and subsistence. The program was pilot-tested at Moi Teaching and Referral Hospital and later introduced in the eye ward of Kenyatta National Hospital. With more families registered with NHIF, which covers inpatient treatment, the financial burden of families associated with treatment has been greatly reduced. Recently, NHIF has been updated to cover the bulk of treatment cost for pediatric cancers. This has lead to a shift in the role of DECF-K and ‘Cycle of Light’ to increase awareness of the existence of NHIF, ease the membership process and assist parents in paying premiums.

#### Research outputs

The KNRbS partnership has yielded 8 collaborative publications in peer-reviewed scientific journals (Additional file 1: Table S1).

Basic, critical retinoblastoma information and insights were collected at the onset of the KNRbS. Three-year survival was found to be 26 % [16]. Probable causes for low survival were identified: children in Kenya present with late-stage retinoblastoma [17], and the time between initial diagnosis and treatment in a referral centre is extensive, and children were lost to follow-up [18]. These baseline calculations will be used as comparison statistics for follow-up research to determine the impact of KNRbS efforts; the studies are currently under way.

A study led by members of the KNRbS genetics task force (a sub-committee of the Medical Care Task Force) identified a need to bolster retinoblastoma genetics education amongst clinicians [19]. The difficulty in communicating complex genetic information is well-established, yet effective dissemination of genetic information is critical for families to be able to make decisions in their best interest, and to improve treatment compliance [20–22]. To address this, an interactive retinoblastoma genetics workshop was designed with the aims of honing cancer genetic counselling skills and improving knowledge of retinoblastoma genetics, including diagnostics, amongst clinicians [23]. Additional research publications in preparation will document outcomes of health service delivery innovations in pathology and child life.

#### Health service delivery innovations

Four health service delivery innovations were piloted by the KNRbS (Additional file 1: Table S1).

### Enucleation technique & artificial eyes

Prior to the KNRbS, children who had an eye removed (enucleation) for retinoblastoma did not receive an implant, the purpose of which is to fill the resultant empty space in the orbit, allow the fitting of a prosthetic eye. This was partly due to a misconception held by physicians that an implant could mask detection of cancer recurrence. Moreover, without an implant, children remain with an empty socket, facing stigmatization and higher infection risk in the orbit. For these reasons, parents found it difficult to accept enucleation even if it was the only cure for their child.

The combination of surgical training provided at the KNRbS meeting introduced the myoconjunctival enucleation technique, which uses an inexpensive implant that was first implemented and studied in India [15]. Training in Kenya helped dispel the misconception related to masking of orbital recurrence, and coupled with the availability of histopathology from the RbCoLab project (see next section), physicians now had a more accurate and powerful approach to predict children who were at risk of recurrence.

Procurement of artificial eyes was another challenge that was solved through coordinated efforts of DECF and individual KNRbS members to purchase low-cost prosthetics from India, and distribute them uniformly to the clinics in the network. Now every child in Kenya can look forward to an artificial eye if needed. This is an example of a so-called ‘South-South’ innovation, as the solution was adapted from another developing country.

### The Retinoblastoma Collaborative Laboratory (RbCoLab)

When retinoblastoma is treated by enucleation, histopathological analysis is essential in order to determine the extent of tumor involvement in the eye. Tumor cells evident in the optic nerve or sclera indicate a greater probability that the tumor has escaped the eye, necessitating further treatment for the child. However, too often this important part of care is either unavailable, or available and slow, incomplete or inaccurate.

The KNRbS group designed a system of centralized pathology for retinoblastoma, called the Retinoblastoma Collaborative Laboratory (RbCoLab) [24]. KNRbS meetings were used to achieve consensus on pathology request and reporting forms. A pathologist was trained in ocular and retinoblastoma pathology, and subsequent training of pathologists and pathology technicians ensued (Additional file 1: Table S1). A specimen collection kit was developed and distributed to eye units where retinoblastoma enucleations were performed, and eyes couriered to RbCoLab for processing. Reports with results were returned to physicians electronically, with digital images of pathology slides available online. An analysis of the impact of RbCoLab is forthcoming.

### eCancerCare-Retinoblastoma

The KNRbS has tested the feasibility of implementing a national retinoblastoma-specific electronic patient database, eCancerCare-Retinoblastoma (eCCRB) [21, 22]. First developed in Canada, eCCRB is a point-of-care medical record that documents patient demographics, treatment and follow-up data using evidence-based guidelines to inform data collection. A national medical record for patients with retinoblastoma allows all doctors in the patient’s circle of care to input and access data, generating an instant, complete picture of patient history and prognosis. In a student project, the KNRbS first tested feasibility of using eCCRB use at multiple retinoblastoma clinics, and offered feedback to Canadian developers on adding data collection fields unique to resource-limited settings. The updated Version 2.0 of eCCRB was then hosted at the University of Nairobi on a nationally accessible server, and user training took place at KNRbS workshops. The group faced initial technical and logistical challenges implementing eCCRB, including lack of network connectivity in some Operating Theatres, limiting the ability to enter data at point-of-care. The group is currently creating work-arounds to these challenges.

### Child life program

Early in the development of the KNRbS, it was recognized that Child Life, a strategy for helping children cope with hospitalization and treatment [25], could dramatically improve care for children with retinoblastoma. Child Life specialists provide psychosocial support and medical education to children and their families, and use simple techniques to help children cope with sometimes traumatizing medical procedures. Child life programs have positive psychological impacts on participants, and can actually lower health care costs by reducing the duration of hospital stays and decreasing use of narcotics for treatment [26]. In a resource-limited setting like Kenya, this latter point becomes extremely relevant.

Child Life principles were introduced to the KNRbS group through a series of annual lectures and practical training sessions taking place the week prior to each KNRbS meeting, led by a Canadian Certified Child Life Specialist (CCLS) KNRbS member. Five KNRbS members representing five key regions in Kenya (Coast, Nairobi, Western, Rift Valley and Nyanza) were selected for training as ‘Child Life Leaders’, and to introduce Child Life practices through patient care in their home institutions. The Sally Test Pediatric Center at Moi Teaching & Referral Hospital in Eldoret hosted these training sessions. In return, members of Moi staff were invited to the training, effectively bringing child life to Kenya beyond retinoblastoma alone. Child Life Leaders kept logbooks of implementation of child life into their daily professional practice, supervised and monitored by their CCLS mentor. The combination of coursework and experiential learning has prepared the trainees to meet the eligibility requirements to write the Child Life Certification Exam, scheduled to take place in Eldoret in September 2015. The will be the first certification examination to take place outside of North America, and pending their success, the trainees will become the first Certified Child Life Specialists in Kenya.

## Discussion

We mapped the achievements of the KNRbS to the KFPE framework (Table 3). The KNRbS fulfills 9/11 principles, with ongoing efforts to strengthen 2/11 principles, namely principles *(10) Apply Results* and *(11) Secure Outcomes* (Table 3).

**Table 3:** Evaluation of the KNRbS by Swiss Commission Principles on Effective International Research Partnerships

Recommendations (KFPE)	Achieved?	Evidence of Achievement	Challenges
1. Set The Agenda Together	Yes	The Situation Analysis performed at the first meeting with the involvement of the entire group highlighted immediate priorities of the strategy and agenda-setting.	Multidisciplinary group has different viewpoints; although agenda is mutually agreed-upon, there may be conflict in the approach to be used.
2. Interact with Stakeholders	Yes	KNRbS meetings are multidisciplinary and include members from multiple sectors.	Establishing a partnership required time; needed to build trust and delineate mutual benefit.
3. Clarify Responsibilities	Yes	Roles and responsibilities are set at KNRbS meetings in person.	Communication challenges outside of the meeting made it difficult to follow-up with roles & responsibilities of members.
4. Account to Beneficiaries	Yes	Survivors & retinoblastoma families are part of the KNRbS group. They serve as moral ‘compass’ to keep KNRbS on track to achieve targets for the benefit of patients, and provide lived experience to influence health service delivery.	Multidisciplinary collaboration is difficult, particularly between lay people and science/medical teams.
5. Promote Mutual Learning	Yes	Multiple workshops and learning opportunities have been offered.	Fellowship funding is limited, yet fellowships provide most intensive form of training.
		Invited speakers that bring different perspectives to the group; promotes cross-talk between people with differing areas of expertise.	
6. Enhance Capacities	Yes	Progress made in training, access to equipment, and developing research capacities.	Different stakeholders had differing expectations for the focus of capacity building, which required a need to clarify priorities.
7. Share Data and Networks	Yes	An interconnected Kenyan referral system for patients has been established. Case studies from collaborating centers discussed at KNRbS meetings	Skills & resources are not always evenly distributed during early capacity building initiatives. For example, most patients are treated in Nairobi, whereas child life initiatives have been developed in Eldoret.
		Informal communication between participants has strengthened relationships and resulted in research collaborations.	
8. Disseminate Results	Yes	Results have been disseminated via publications, conferences, and awareness materials and media.	Participation at international meetings difficult due to limited funding.
		Consensus guidelines have been produced and made available to all Kenyan professionals, and online.	
9. Pool profits and merits	Yes	Many grant applications were successfully funded.	Few funding sources are available in Kenya.
		Research is increasingly conducted by Kenyan investigators and trainees.	Under-developed infrastructure for receiving grants in Kenya keeps group dependent on foreign partners.
		There is a transparent system of authorship on publications.	
10. Apply Results	Yes - ongoing	Health service delivery innovations are being pilot tested.	Funding limited to support these initiatives; need to consider innovative approaches to ensuring sustainability of efforts.
		An assessment of barriers to successful implementation performed; facilitators of implementation identified.	
11. Secure Outcomes	Yes - ongoing	Partnership with civil society and government is intended to secure the sustainability of the gains in retinoblastoma outcomes.	Establishing partnerships required time; needed to build trust and delineate mutual benefit.
		Endorsement of KNRbS guidelines by Kenyan Ministry of Health validates recommendations.	Progress undermined by changes in political stability, university and public hospital strikes, etc.
		Securing outcomes for retinoblastoma requires showing relevance of KNRbS approach to other childhood cancers.	

With respect to *(10) Apply Results*, we have shown how KNRbS activities have produced consensus national guidelines for care, and supported capacity-building initiatives to facilitate their implementation. The group is on track to evaluate the outcomes of their activities, such as effect on availability, accessibility and quality of care, and ultimately on patient survival at the national level. Assessing barriers and facilitators to implementation will provide information on reach and scalability of efforts, such that no child is left behind (Table 3).

Moreover, *Securing Outcomes* (Principle 11) in the long-term is necessary for sustained effect of KNRbS activities on the care, survival and well-being of children with retinoblastoma. For this principle, the KNRbS will leverage partnerships within government and civil society to sustain its impact (Table 3). One step in this direction is the amalgamation of DECF-K within the Kenyan Childhood Cancer Trust (KCCT), an organization devoted to improving care and survival for all pediatric cancers. Inspired by the KNRbS national strategy approach, the KCCT will learn from the retinoblastoma experience and effect similar impact on other childhood cancers.

We noted 3 key features of the KNRbS partnership that were essential to produce the results we observed: 1) Multidisciplinarity, 2) Consistency, and 3) Flexibility.

The integration of multiple, distinct disciplines into the KNRbS group made many key initiatives possible by leveraging disparate skills and expertise. This included administrative and diplomacy strengths from DECF members; scientific expertise for experimentation and measurement of outcomes; academic expertise for the design and delivery of training programs; medical and psychosocial care expertise for innovation in healthcare delivery; and the lived experience of retinoblastoma, which influenced all spheres of KNRbS work, and united members to a common purpose. The definition of global health, as a discipline, has a focus on issues of health equity with a multidisciplinary approach [22].

A major challenge of working within a multidisciplinary team such as the KNRbS however, is the inevitable conflict that arises based on discipline-specific biases [21]. Although the KNRbS agenda and overarching goals were established collectively as a group, the approaches suggested to meet those goals varied widely between disciplines. For example, civil society members were more prone to supporting business-minded principles in program design, in contrast to researcher members who valued unstructured tinkering and experimentation.

The consistency of annual meetings ensured a dependable forum to openly discuss different perspectives, and work towards building consensus on solutions. The regular interactions helped build trust among group members, and elicit personal investment and accountability in KNRbS efforts. While the group still faces barriers in working together (for example, the institutional barriers that may preclude a national approach for some projects), the consistency of the meeting ensures that a forum exists in which to discuss these barriers and explore plausible solutions.

Consistency in the KNRbS has been especially crucial in the success of coordinating this sizable, geographically dispersed and diverse team. The KNRbS was conceived as a national strategy, which stems naturally from the multidisciplinary, Kenya-wide expertise required for best care for retinoblastoma (for example, while enucleations can be performed at several secondary treatment centers, radiotherapy and focal therapy are only available at one teaching hospital [18]). However, orchestrating a coherent national strategy from inception has presented logistical challenges including several mentioned previously (eg. technical and communication challenges). Coordination of national strategies with local initiatives was identified as a key challenge in the strategic management of national strategies [27]. These difficulties have been largely offset with an exquisite balance of consistency and dependability from the annual meeting.

Finally, the third key element contributing to the success of the KNRbS is flexibility. The evolution of core focus from capacity building to research and innovation demonstrates this ideal. Flexibility is evident in the changing timelines and approaches in producing Best Practice Guidelines and the current focus of the group on addressing challenges with implementing eCCRB. In addition, flexibility was key for the Cycle of Light program: when circumstances changed such that families could readily access NHIF, a shift in focus from aiming to pay for treatment to facilitating NHIF registration occurred. However the lessons learned from the program, working with patients, and connections made in the hospital beyond just the KNRbS, reaped benefits that were re-invested into new KNRbS endeavors.

The achievements of the KNRbS have been facilitated by key technological advancements. For example, RbCoLab has permitted access to accurate pathology for retinoblastoma cases in disparate locations where a retinoblastoma-specialized pathologist is not available. Accurate pathology is necessary for successful retinoblastoma treatment, yet the number of pathologists in Kenya is acutely limited [24]. The ability to rapidly communicate findings electronically is critical for timely retinoblastoma treatment. Similarly, connectivity is central to eCCRb, which will organize and ease access to patient data nationwide. The crux of the benefits of technology is that it has permitted the communication and organization of retinoblastoma management over vast distances nearly immediately.

The KNRbS group has achieved a number of key outputs with relatively little financial investment from its inception. This underscores the value of having a committed group with a shared vision, despite having few financial resources. Progress has been made incrementally, with each success setting the stage for growth. The achievements of the KNRbS group speak to the value of the intellectual capital provided by multidisciplinary networks such as this.

## Next steps

Currently in the Innovation, Research, and Implementation Phase, efforts of the KNRbS will focus on implementing results and securing their effects for maximum sustainability of the program. Long-term patient outcomes will be studied at the national level in Kenya. Within greater East Africa, the KNRbS has inspired national strategies in Uganda and Ethiopia. Delegates from these countries have attended past KNRbS meetings, and have hosted their own national retinoblastoma meetings with guidance from KNRbS since 2013.

The KNRbS group will continue to be dynamic. For example, the KNRbS NGO partners are evolving in their roles. DECF has rebranded itself as World Eye Cancer Hope, retaining its mission to save vision and lives globally, but using the experiences from Kenya, Canada, US and UK to guide its work on a broader international scale. The Kenyan chapter of DECF, now absorbed by the KCCT, will guide the application of the national strategy approach improve survival from other pediatric cancers, using the lessons learned from retinoblastoma in Kenya as a model.

## Conclusions

The KNRbS was initiated in Kenya in 2008 in response to the poor outcomes for children with an otherwise curable eye cancer. Knowledge that optimal care required a multidisciplinary approach influenced the membership of the group. Although resource mobilization was essential, it was not the overarching focus of the group, and a large number of outputs was achieved with careful design, leveraging of skills and networks of multisectoral members.

## Abbreviations

DECF, Daisy’s Eye Cancer Fund; DECF-K, Daisy’s Eye Cancer Fund-Kenya; KNRbS, Kenyan National Retinoblastoma Strategy; eCCRB, eCancerCare-Retinoblastoma; NHIF, National Health Insurance Fund; KFPE, Swiss Commission for Research Partnerships with Developing Countries.

## Additional file

Additional file 1: KNRbS Task Forces & Achievements. (DOCX 5222 kb)
