# Supplementary material for: Achieving optimal cancer outcomes in East Africa through multidisciplinary partnership: a case study of the Kenyan National Retinoblastoma Strategy group
Source: Global Health. 2016 May 26;12:23. doi: 10.1186/s12992-016-0160-1 (PMC4882853; doi:10.1186/s12992-016-0160-1)
Supplement: Additional file 1: — KNRbS Task Forces & Achievements. (DOCX 5222 kb) [file 12992_2016_160_MOESM1_ESM.docx]

| **1° Task Force** | **2° Task Force** | **Output Type** | **Output Subtype** | **Year** | **Name of Output** | **Journal / Media Outlet / Implementor** | **URLs** |
| --- | --- | --- | --- | --- | --- | --- | --- |
| Family Support |  | Health Service Delivery Innovation | Psychosocial Support | 2008 | Child Life Program | Daisy's Eye Cancer Fund-Kenya/Sally Test Pediatric Center |  |
| Family Support | Awareness | Film | Documentary | 2014 | Daisy Fund Brings Child Life to Kenya | Moi Teaching & Referral Hospital | <https://www.youtube.com/watch?v=0B_Wu2Upp3I> |
| Awareness |  | Policy/Guidelines | Mother & Child Health Booklet | 2011 | Insert on Retinoblastoma Detection | Ministry of Health, Kenya |  |
| Awareness |  | Non-peer reviewed publication | NGO Report (incl. KNRbS Situation Analysis) | 2008 | Rati's Challenge: A Vision for Africa | Daisy's Eye Cancer Fund |  |
| Awareness |  | Non-peer reviewed publication | Academic Report | 2008 | The Kenyan National Retinoblastoma Strategy: Building local capacity in the diagnosis and management of pediatric eye cancer in Kenya | University of Toronto Ophthalmology Rounds |  |
| Awareness |  | Print Media | News Article | 2009 | Is that a trick of the light or cancer? | Daily Nation | <http://www.nation.co.ke/lifestyle/Living/-/1218/562450/-/2jf40a/-/index.html> |
| Awareness |  | Print Media | News Article | 2014 | Helping solve Kenya's retinoblastoma challenge | University of Toronto News | <http://news.utoronto.ca/helping-solve-kenyas-retinoblastoma-challenge> |
| Awareness |  | Print Media | News Article | 2009 | Alert raised over child eye cancer | Daily Nation | <http://www.nation.co.ke/news/-/1056/644844/-/4eybifz/-/index.html> |
| Awareness |  | Radio | Live Radio Call-in show | 2011 | Awareness & Call-in show with KNRbS Members | Baraka FM, Mombasa Kenya |  |
| Awareness |  | TV | News Show | 2011 | Beads against rise in cancer cases | Video Journalist Africa | <https://www.youtube.com/watch?v=rUbWTMbJVlI> |
| Awareness |  | TV | News Show | 2010 | Health Digest segment on retinoblastoma | Kenya Television Network Health Digest | <https://www.youtube.com/watch?v=WnhdI6r-RN8> |
| Awareness |  | TV | News Show | 2011 | Tackling Cancer at KNH | Kenya Citizen TV | <https://www.youtube.com/watch?v=HNgoC5w3KO0> |
| Awareness |  | TV | Documentary | 2014 | Retinoblastoma: a children's eye cancer | Masseon Media Centre | <https://www.youtube.com/watch?v=MjzTdY0IIk4> |
| Awareness |  | Conference presentation | Oral presentation | 2015 | Global Genetics: A Novel Approach to the Delivery of Retinoblastoma Genetic Services | International Society of Ocular Oncology |  |
| Awareness |  | Conference presentation | Oral presentation | 2014 | Global Retinoblastoma Genetics: Sharing the Care Worldwide | One Retinoblastoma World |  |
| Awareness |  | Conference presentation | Poster presentation | 2015 | Cancer Genetics Education in a Low- to-Middle-Income Country: Evaluation of an Interactive Workshop for Clinicians in Kenya | Consortium of Universities for Global Health |  |
| Awareness |  | Conference presentation | Oral presentation | 2011 | The Global Retinoblastoma Clinic | International Society for Eye Diseases and Retinoblastoma |  |
| Medical Care | Awareness | Policy/Guidelines | Best Practice Guidelines | 2014 | Retinoblastoma Best Practice Guidelines | Ministry of Health, Kenya | <http://guidelines.health.go.ke/#/category/6,7/4/meta> |
| Medical Care |  | Peer-Reviewed Scientific Journal Article | Original Article | 2015 | Cancer Genetics Education in a Low- to-Middle-Income Country: Evaluation of an Interactive Workshop for Clinicians in Kenya | PLOS ONE |  |
| Medical Care |  | Peer-Reviewed Scientific Journal Article | Letter | 2012 | The incidence and distribution of retinoblastoma in Kenya | British Journal of Ophthalmology |  |
| Medical Care |  | Peer-Reviewed Scientific Journal Article | Short Communication | 2014 | Developing Clinical Cancer Genetics Services in Resource-Limited Countries: The Case of Retinoblastoma in Kenya | Public Health Genomics |  |
| Medical Care |  | Peer-Reviewed Scientific Journal Article | Original Article | 2014 | Retinoblastoma Referral Pattern in Kenya | Middle East & Africa Journal of Ophthalmology |  |
| Medical Care |  | Peer-Reviewed Scientific Journal Article | Editorial | 2013 | Digital cancer pathology in Africa | Lancet Oncology |  |
| Medical Care |  | Peer-Reviewed Scientific Journal Article | Review | 2012 | Retinoblastoma | Lancet |  |
| Medical Care |  | Peer-Reviewed Scientific Journal Article | Editorial | 2011 | Challenging the global retinoblastoma survival disparity through a collaborative research effort | British Journal of Ophthalmology |  |
| Medical Care |  | Peer-Reviewed Scientific Journal Article | Original Article | 2013 | Survival among retinoblastoma patients at the Kenyatta National Hospital, Kenya | Journal of Ophthalmology of Eastern, Central and Southern Africa |  |
| Medical Care |  | Health Service Delivery Innovation | Pathology | 2012 | Retinoblastoma Collaborative Laboratory | University of Nairobi |  |
| Medical Care |  | Health Service Delivery Innovation | Surgery | 2013 | Enucleation & Artificial Eyes | Daisy's Eye Cancer Fund-Kenya/KNRbS |  |
| Medical Care |  | Health Service Delivery Innovation | Medical Care | 2009 | eCancerCare-Retinoblastoma: electronic patient record management | University of Toronto/KNRbS |  |
| Medical Care |  | Training | Observership/Fellowship | 2009 | Ocular & Retinoblastoma Pathology | University of Toronto/The Hospital for Sick Children |  |
| Medical Care |  | Training | Observership/Fellowship | 2013 | Clinical Retinoblastoma Training | University of Toronto/The Hospital for Sick Children |  |
| Medical Care |  | Training | Workshop | 2012 | Pathology Technician Training: Eye Processing | University of Nairobi |  |
| Medical Care |  | Training | Workshop | 2013 | Retinoblastoma Genetics Interactive Workshop | University of Toronto/University of Nairobi/KNRbS |  |
| Medical Care |  | Training | Workshop | 2012 | Pathologist & Clinician Training | University of Toronto/University of Nairobi/KNRbS |  |
| Medical Care |  | Training | Workshop | 2013 | Pathologist & Clinician Training | University of Toronto/University of Nairobi/KNRbS |  |
| Medical Care | Resource Mobilization | Grant | Meeting, Planning, Dissemination Grant | 2008 | The 1st Kenyan National Retinoblastoma Strategy Group Meeting | Canadian Institutes of Health Research |  |
| Medical Care | Resource Mobilization | Grant | Meeting, Planning, Dissemination Grant | 2011 | The 4th Annual Kenyan National Retinoblastoma Strategy Group Meeting | Canadian Institutes of Health Research |  |
| Medical Care | Resource Mobilization | Grant | Global Health Innovation Grant | 2012 | Cancer Pathology in Africa | Grand Challenges Canada |  |
| Medical Care | Resource Mobilization | Grant | Meeting Grant | 2012-2014 | KNRbS Annual Meetings (2012-2014) | TUYF Charitable Trust |  |
| Medical Care | Resource Mobilization | Grant | Meeting Grant | 2009-2010 | KNRbS Annual Meetings (2009-2010) | Kalmar Family Foundation |  |
| Resource Mobilization |  | Fundraiser | NA | 2014 | Africa Bush Trek | Daisy's Eye Cancer Fund - International | <https://www.youtube.com/watch?v=h1iFQIxyuhY> |
| Resource Mobilization |  | Fundraiser | NA | 2012 | Bamburi Treasure Hunt | Daisy's Eye Cancer Fund - Kenya |  |
| Resource Mobilization |  | Fundraiser | NA | 2009 | Toronto Marathon | Daisy's Eye Cancer Fund - Kenya & Canada |  |
